# Sentimental text mining based on an additional features method for text classification

**DOI:** 10.1371/journal.pone.0217591

**Published:** 2019-06-05

**Authors:** Ching-Hsue Cheng, Hsien-Hsiu Chen

**Affiliations:** Department of Information Management, National Yunlin University of Sci. & Tech., Douliou, Yunlin, Taiwan; Politechnika Krakowska im Tadeusza Kosciuszki, POLAND

## Abstract

Owing to the emergence of the Internet and its rapid growth, people can use mobile devices on many social media platforms (blogs, Facebook forums, etc.), and the platforms provide well-known websites for people to express and share their daily activities and ideas on global issues. Many consumers utilize product review websites before making a purchase. Many well-known websites are searched for relevant product reviews and experiences of product use. We can easily collect large amounts of structured and unstructured product data and further analyze the data to determine the desired product information. For this reason, many researchers are gradually focusing on sentiment analysis or opinion exploration (opinion mining) and use this technique to extract and analyze customer opinions and emotions. This paper proposes a sentimental text mining method based on an additional features method to enhance accuracy and reduce implementation time and uses singular value decomposition and principal component analysis for data dimension reduction. This study has four contributions: (1) the proposed algorithm for preprocessing the data for sentiment classification, (2) the additional features to enhance the accuracy of the sentiment classification, (3) the application of singular value decomposition and principal component analysis for data dimension reduction, and (4) the design of five modules based on different features, with or without stemming, to compare the performance results. The experimental results show that the proposed method has better accuracy than other methods and that the proposed method can decrease the implementation time.

## Introduction

The volume of data from social media and online activities (e.g., chat rooms, e-commerce, and blogs) is classified as big data, which allow us to easily collect a large amount of structured and unstructured data. To find valuable information, we must extract and analyze the collected data, and this trend refers to big data. Many researchers have proposed automatic text categorization and data analysis methods; these techniques include data mining, web mining, and text mining. The datasets regarding customers’ opinions or reviews are often massive and hard to analyze; it requires additional approaches to summarize them. Many forums, product marketing websites, mobile applications, e-commerce websites, and related web resources have provided platforms for consumers to express their opinions. These consumer opinions could be studied to determine the public opinions and the behavioral trends of consumers for the strategies and marketing campaigns of companies, product preferences of consumers, and for monitoring reputations [1]. Review platforms have become more prevalent, and they are important resources for extracting and analyzing opinions. In addition, a customer who wants to buy a product will often look for information on the Internet to find relevant opinions; therefore, analyzing reviews has become increasingly important in the real world. Sentiment analysis (SA) can be used to analyze people’s opinions, sentiments, emotions, and attitudes expressed in texts [2]. In many fields, sentiment analysis has achieved good results, especially in intelligent marketing [3], customer satisfaction [4], and sales prediction [5]. Moreover, it is a challenge to find the efficient features for representing the text.

In general, it is impossible for users to read all the reviews from the various review resources. Based on a brief summary, many researchers have been working on sentiment analysis for a long time. Sentiment analysis is the study of the computational methods for extracting the opinions, sentiments, emotions, and attitude expressed in texts towards an entity [2]. Sentiment analysis, also called opinion mining, sentiment mining, review mining or attitude analysis, is the task of detecting, extracting, and classifying opinions. In addition, sentiment analysis is focused on the different issues which are addressed in the review or text [6].

In text mining, there are two main parts: (i) extracting and selecting features, and (ii) utilizing an algorithm for classification. In extracting and selecting features, [7] employs unigrams, bigrams, and parts of speech (POS) to denote movie reviews. In addition, [8] represents their data using n-gram sequences with POS tagging. In terms of classification, [9] trains their classifier by inputting the matrix that transforms the data and using a TF-IDF method. Finding the relevant features for treating a text is very challenging. Because reviews usually contain less than 300 words, it is hard to find the features that represent the entity. In addition, [10] shows that many works do not have uniform experimental settings. To address these issues, this paper proposed additional features and an “SVD then PCA” method to enhance accuracy and reduce implementation time for text mining, and, based on stemming, designs five module experiments with different features to compare performance and explore what factors affect the classification accuracy. In summary, the objectives of this study are as follows:

1. Present a sentimental text mining method based on an additional features method to enhance the classification accuracy of big data analysis of sentiment reviews;
2. Propose a feature extraction algorithm to increase the accuracy of sentiment classification;
3. Utilize an efficient “SVD then PCA” method to reduce the data dimensionality and implementation time.

This paper is organized as follows. Section 2 presents related works, including product reviews, sentiment mining, feature extraction and selection, SVD and PCA methods, and classifiers. The research concept and proposed method are introduced in section 3. Section 4 presents the experimental results. Finally, the conclusion is presented in section 5.

## Materials and methods

### Related literature

This related literature and concepts, including product reviews, sentiment mining, feature extraction and selection, and classifiers are introduced briefly in the following sections.

#### Product reviews

The online review of products is provided by a website which publishes consumer opinions on products, services and businesses. Due to Web 2.0, many people use electronic word-of-mouth to post their experiences and preferences for various products. On-line product reviews deliver more accessible information to enterprises for understanding the perceptions and preferences of consumers. Many previous studies on sentiment mining collected product reviews to analyze product properties because consumers review the related information to determine whether to buy the product or not, and a decrease in the quantity of product information could help consumers make decisions. Indeed, reviews were seen as a diagnostic tool for reducing the uncertainty of purchasing a product [11]. [12] proposed an econometric preference measurement model to extract consumers’ preferences from online product reviews. Furthermore, Archak, Ghose, & Ipeirotis [13] revealed that the review opinions of customers are useful for enterprise strategies.

#### Sentiment mining

Sentiment analysis is a popular application in text analytics that employs data analysis on the text to understand the expressed opinions. Subjective text is usually conveyed by humans with typical moods, emotions, and feelings. SA is widely used, especially in social media analyses, and includes many techniques to implement natural language processing (NLP), information retrieval (IR), and structured/unstructured data mining. The main challenge is that real world data are unstructured [10]. There have been many research efforts in recent years to obtain important and useful information from these unstructured datasets. From the work of [10], sentiment analysis can be divided broadly into six tasks as follows:

1. Subjectivity classification [7];
2. Sentiment classification [10, 14–16];
3. Review usefulness measurement [17];
4. Lexicon creation [18];
5. Opinion spam detection [10]; and.
6. Opinion word and product aspect extraction [2, 10].

From the literature, data acquisition and preprocessing is the first step in sentiment mining, and this important step affects the whole process. The second step is to extract the features from the raw data and apply a machine learning method for classification. Therefore, this study summarized the reviews of sentiment mining for different categorization schemes and techniques, as shown in Table 1.

**Table 1:** Reviews of the sentiment mining for different categorization schemes and techniques.

Authors	Categorization	Techniques
**Saleh, Martin-Valdivia, Montejo-Ráez, & Ureña-López (2011) [1]**	Polarity determination	SVM
**Turney (2002) [19]**	Polarity determination	PMI-IR
**Pang, Lee, & Vaithyanathan (2002) [7]**	Polarity determination	NB, SVM, ME
**Li & Tsai (2013) [14]**	Vagueness in opinionated text	FFCA, Lattice
**Moraes, Valiati, & Neto (2013) [9]**	Polarity determination	NB, SVM, NN
**Li & Li (2013) [3]**	Applications of SA	SVM, NB
**Bollegala, Weir, & Carroll (2013) [16]**	Cross-domain SA	Cosine similarity, L1 regularized logistic regression
**Abbasi, France, Zhang, & Chen (2011) [20]**	Polarity determination	Rule-based multivariate features, SVM
**Tan, Cheng, Wang, & Xu (2009) [15]**	Cross-domain SA	Adaptive-NB
**Rui, Liu, & Whinston (2013) [5]**	Applications of SA	SVM, NB
**Whitelaw, Garg, & Argamon (2005) [24]**	Polarity determination	DBA, SVM, and SMO-SVM
**Kang & Park (2014) [25]**	Applications of SA	DBA
**Pang & Lee (2004) [21]**	Polarity determination	Minimum Cuts
**Parmar, Bhanderi, & Shah (2014) [22]**	Polarity determination	RF
**Nigam, Lafferty, & McCallum (1999) [29]**	Polarity determination	ME
**Rahate and Emmanuel (2013) [8]**	Polarity determination	SVM

#### Feature extraction and selection

Feature extraction and selection have been widely discussed and analyzed in text mining for a long time. The aim of feature extraction is to represent documents as multidimensional vectors [23]. Feature selection or feature extraction techniques are employed to reduce the dimensionality of the corpus and improve the training time of the classifier. Feature extraction is used to extract new features by some functional mapping from all feature sets [24]. The critical problem of feature extraction is that when the extracted features have no meaning it is hard to interpret their outputs [20].

Feature selection makes the classifier more efficient by reducing the dimensionality of the corpus without reducing its accuracy. Many unsupervised feature selection methods have been proposed in the literature. The most popular methods are the document frequency (DF), term frequency inverse document frequency (TFIDF), term contribution (TC), term variance (TV), information gain (IG), mutual information (MI), and so on. Information gain has been shown to be more competitive than the other methods [5, 20].

#### Singular value decomposition

In the area of linear algebra, singular value decomposition (SVD) is a reduced matrix computation, the eigenvalue decomposition can only be utilized on square matrices. The SVD technique is used when researchers want to obtain the eigenvalues and eigenvectors for a matrix [25]. That is, matrix A is factorized into the product of three matrices A = UDV^T^, where U and V are orthonormal and matrix D is diagonal with a positive real number. SVD has been applied in many fields; in many cases, matrix A is close to a low rank matrix which can be determined and which is a good approach to the data matrix., i.e., we can obtain matrix B of rank k, which is the best matrix close to A; in fact, we can try every k for different applications. Furthermore, SVD is defined for all matrices (rectangular or square) unlike many commonly used spectral decomposition method in linear algebra. In SVD, the eigenvalues can be employed as decision criteria to determine the matrix size for data dimension reduction.

#### Principle component analysis

Principle component analysis (PCA) [26] is a dimension reduction technique that can be employed to reduce a large set of variables to a small set such that the selected principal components retain most of the information from the original data. PCA is a statistical computation that transforms the correlated variables into a smaller number of uncorrelated principal components (PC). The first principal component accounts for most of the variability in the data, and each succeeding component accounts for as much of the remaining variability as possible. PCA is similar to factor analysis in multivariate statistics. In general, the number of components is smaller than the number of original variables in the data. PCA can be explained as fitting an n-dimensional ellipsoid to the data where each axis of the ellipsoid denotes a principal component. The covariance matrix of the data and the eigenvalues and corresponding eigenvectors of the matrix will be computed and calculated. Finally, the set of eigenvectors must be orthogonalized and normalized to unit vectors. Both SVD and PCA are global algorithms that can extract the main features of a dataset. PCA is focused more on the covariance matrix, whereas SVD is focused more on the data itself [27].

#### Machine learning classifiers

This study chose four popular classifiers; these classifiers are mostly employed in sentiment classification. The four classifiers are naïve Bayes (NB), support vector machines (SVM), maximum entropy (ME), and random forest (RF). Next, the four classifiers are introduced as follows.

**Naïve Bayes.** The naive Bayes (NB) classifier [28] is based on Bayes’ theorem and is particularly appropriate when the dimensionality of the inputs is high as it is a simple probabilistic classifier. From the basic Bayes’ theorem, consider the probability of a particular document, d, being assigned to a class, c_i,_ and x_i_, which is an individual word of the particular document. Then, P(c_j_) and P(x_i_|c_j_) are calculated from the training data, and P(x_i_|c_j_) is also the conditional probability of x_i_ appearing in a document of class c_j_. Although it is a simple method with a conditional independence assumption that cannot capture real-world situations, its advantages are simple and it has surprisingly good accuracy [28].

**Maximum entropy.** Maximum entropy (ME) is a useful tool in several NLP fields [29] that can be utilized to estimate any probability distribution. ME has been verified to be a viable and competitive algorithm in text classification. The ME principle is that when nothing is known, the distribution should be assumed to be uniform. This study is interested in ME classification which is sometimes better than naïve Bayes for text classification [30]. ME tries to find the parameters that maximize the likelihood of all the training data. [29] mentioned that the ME estimate of P(c|d) is an exponential form.

For example, the function will be triggered if the term “happy” appears and the sentiment of document is positive. The ME classifier is a probabilistic classifier that is a type of exponential model. Unlike the NB classifier, the ME classifier does not assume that the features are conditionally independent of each other. The ME classifier can solve the variant problems of text classification, such as language detection, topic classification, sentiment analysis, and so on.

**Support vector machine.** Support vector machines (SVMs) [31–32] find a hyperplane in an n-dimensional space that clearly classifies the data points. To divide the different classes of data points, there are many possible hyperplanes that could be selected, and the objective is to obtain the hyperplane that has the maximal margin. Support vectors are data points that approach the hyperplane and impact the position and orientation of the hyperplane; support vectors are used to maximize the margin of the classifier. Omitting the support vectors will alter the position of the hyperplane because these points help us establish the SVM. To promote the power of SVM text classification, texts must be transformed into vectors. In a text document, let *c*_*j*_ ϵ {1, −1} (correspondingly positive and negative) be the class of document *d*_*j*_, from the Lagrange multipliers for the SVM, use the derivative of the primal parameters, w, to get its solution [31]. If the data points cannot be partitioned well, the data points will be transformed into a higher dimension to find a separable hyperplane by using a kernel function.

**Random forest.** Random Forest (RF) is a flexible and easy machine learning method [33]. RF is also one of the most useful algorithms because of its simplicity and because it can be used for both classification and regression tasks. The RF classifier averages multiple decision trees from random samples of the database. A decision tree partitions the dataset into smaller subsets and simultaneously builds the tree with decision nodes and leaf nodes. The random forest averages all trees to build a model with lower fluctuations. The RF can run on large datasets efficiently and handle a great deal of variables without deleting variables. The RF classifier employs the bagging and bootstrapping concept [33]; hence, the advantages of RF classifiers are: (1) they reduce overfitting by averaging multiple trees; (2) low variance: multiple trees can be applied to reduce instability in classifier performance where there are different classifications between the training and test data.

### The proposed method

The goal of sentiment classification is to classify a document, text, or review into categories that are already labeled (e.g., positive, negative, happy, sad). The most challenging work for sentiment classification is how to improve the accuracy of the result. Because many factors can affect the analysis, such as different preprocessing steps, the level of the sentiment classification (document or sentence), various features, lexicons, and distinct machine learning methods. In previous works, many studies have shown the differences in the results for feature selection techniques, such as unigrams, bigrams, POS tagging [8], n-gram sequences with POS tagging [7], and TF-IDF [9]. Ravi & Ravi [10] showed that many studies do not have the same experimental setting; hence, this paper was based on Cheng [34] to extend the experiments on additional features for enhancing accuracy and apply the “first SVD then PCA” method for dimension reduction and shortening the running time for text classification. Furthermore, this study utilizes stemming to design five module experiments with different features to compare their performance and discover the factors that affect the classifier accuracy.

**Fig 1:**
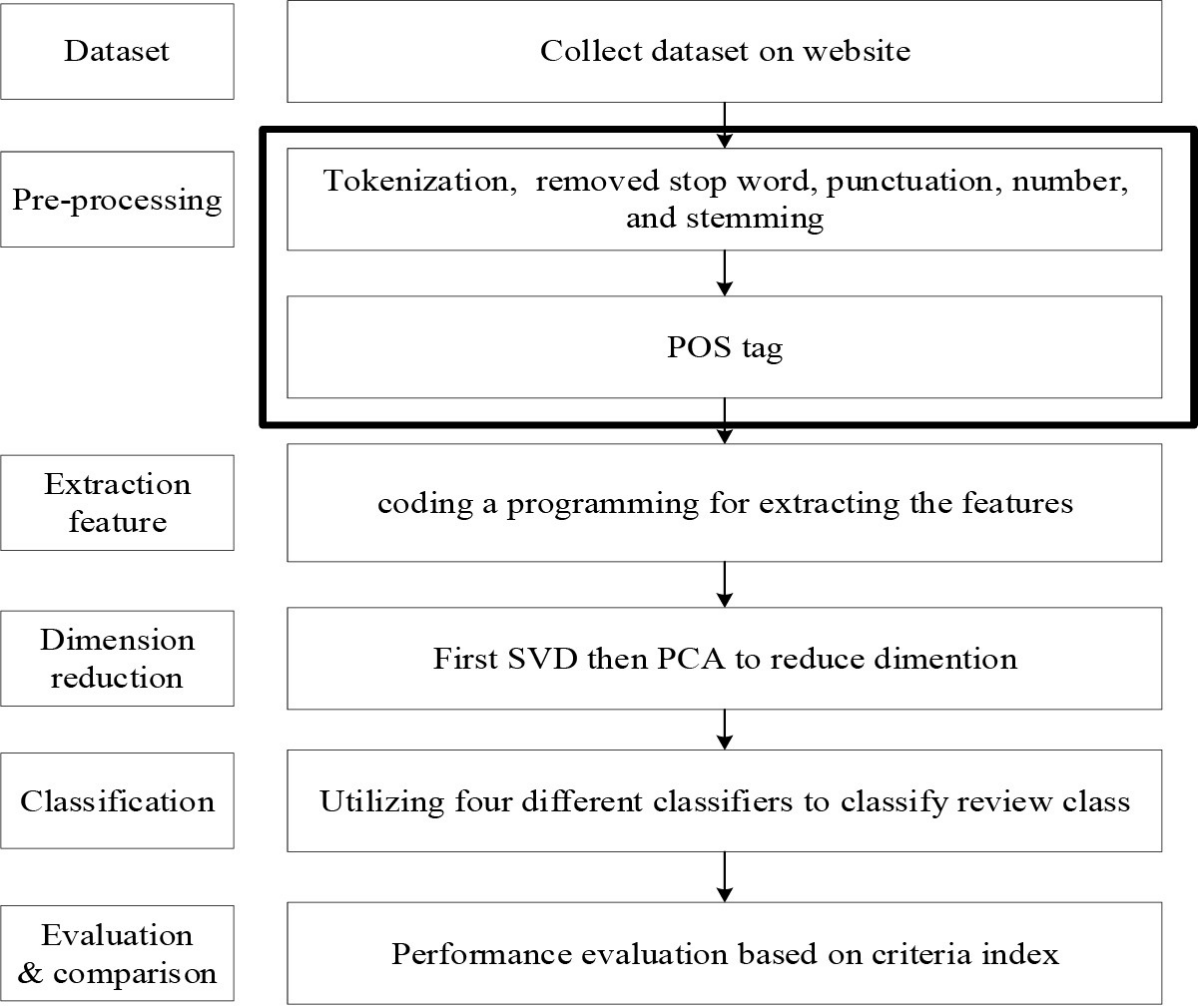
The proposed method.

The procedure of the proposed method is shown in Fig 1. First, the collected dataset is employed for sentiment classification. Second, the preprocessing steps of tokenization, removed stop word, and POS tagging by R statistics are taken. Third, features are defined and extracted, including term frequency–inverse document frequency (TF-IDF), the sentiment score of each document, positive and negative frequencies and the number of adjectives and adverbs. Fourth, the classifier is used to train and predict the data. Finally, the results are evaluated.

#### Proposed algorithm

To easily understand the proposed method, we employ the collected data and present the five main steps to show the computational procedure of the algorithm.

**Step 1 Dataset collection.** First, we collected the most commonly utilized dataset, the Movie dataset [22, 35], which consists of sentimental documents; the Movie review text is not easier to classify than other review texts. The dataset includes 1000 positive and 1000 negative sentiment reviews. We coded an Excel VBA (Microsoft) program to import the text file, and then the labeled sentiment documents were transformed into the MS Excel format.

The second dataset was collected from the OHSUMED dataset created by Hersh et al. [36–37]. The dataset contains 23 different cardiovascular disease categories. The classes C02, 10, 11, 14, and C20 are selected in the experiment because the five classes are related to peripheral nervous system blood vessels. The name of classes and number of features are shown in Table 2.

**Table 2:** OHSUMED category descriptions.

Diseases	Class	Feature
Virus Diseases	C02	391
Nervous System Diseases	C10	1562
Eye Diseases	C11	364
Cardiovascular Diseases	C14	2550
Immunologic Diseases	C20	1220

**Step 2 Preprocessing.** In general, the data collected from the source contain noise; the collected data always need to be processed by several steps before implementing various machine learning methods. This step has five preprocesses, including tokenization, stop word removal, stemming, POS tagging, feature extraction and manifestation [10]. The purpose of tokenization is to remove the punctuation marks in the text. These marks do not contribute to the accuracy of the classifier. Stop words are words we often used in an article, viz., a, the, an and so on. These words do not make the results better, and they sometimes degrade the results. Stemming reduces a word to its root form and ignores the POS of the word. Parts of speech tagging is the process used to identify the different parts of speech of words in the text. Because the data often involves noise, feature extraction is required to help researchers obtain the relevant information. This step used two R language packages called RTextTools and openNLP to process the POS. Feature extraction will be discussed in the next subsection. Apart from feature extraction, feature selection is also an important step, which will certainly affect the analysis result.

**Step 3 Feature extraction and additional features.** The study defined a feature set including the TF-IDF, frequency of positive terms, frequency of negative terms, frequency of adjectives, and frequency of adverbs, as shown in Table 3. This step converted all the documents into a matrix of TF-IDF weights, and at the same time, let the positive and negative frequencies form another feature set. Next, we utilized POS tagging to count the number of adjectives and adverbs, and then the additional features were added. Table 4 presents the TF-IDF parameter descriptions, and the proposed feature extraction algorithm is shown in Algorithm 1.

**Table 3:** Feature descriptions.

Feature	Description
**TF-IDF**	Utilize unigram to convert the collected text into TF-IDE, the range is 0 to 1.
**Freq. of positive**	Count the positive terms in a document.
**Freq. of negative**	Count the negative terms in a document.
**Freq. of adjective**	Count the terms for POS is adjective.
**Freq. of adverb**	Count the terms for POS is adverb.

**Table 4:** TF-IDF parameter descriptions.

Parameters	Description
**removeSparseTerms**	The terms of matrix are deleted; they have the lowest sparse percentage for empty elements.
**minDocfreq**	words appear less than the set number in documents are discarded for the term-document matrix.
**stemWords**	A logical parameter, use the language specified in language to specify whether stem words

**Algorithm 1**. **Feature extraction**.

**Notation:** T_i_: *ith* text in M. M: a matrix with text and sentiment labeled. PL: lexicon with positive word. NL: lexicon with negative word. W_i_: word list of *ith* text. PM: the terms reveal in both T_i_ and PL. NM: the terms reveal in both T_i_ and NL. Tag: A matrix contains the terms with commentated POS.

**Output:** TF-IDF, PM, NM, frequency of adjective and adverb

1. For each text T_i_ in M:
  - discriminate each T_i_ into unigram and save as W_i_.
  - Match the W_i_ with PL and NL, then turn into PM and NM.
  - Return PM, and NM.
2. For each text T_i_ in M:
  - Annotate the T_i_ with word_token_annotator by using the package openNLP.
  - Annotate the T_i_ with POS after T_i_ is annotated by using word_token_annotations.
  - Count the number of adjective and adverb in Tag.
  - Return the result.

**Step 4 Dimension reduction.** Because the TF-IDF matrix is a large sparse matrix with many zero elements, it is difficult to analyze the matrix. Hence, this step employed the “SVD then PCA” method for dimension reduction of the matrix. After feature extraction, the preprocessed matrix was used as SVD input. The SVD technique was used to decompose the TF-IDF matrix such that the values close to zero were transformed to zero. Then, the PCA technique was applied to process the reduced matrix to decrease the matrix dimensions even further. The output of the PCA is shown in Table 5. Lastly, after reduction, the Movie dataset is reduced from a 2000*46467 vector space to a 2000*2000 vector space.

**Table 5:** Description of PCA outputs.

Output	Description
**Sdev**	the standard deviations of the PC
**Rotation**	the matrix of feature loadings
**Center**	the means of feature
**Scale**	the standard deviations of feature (the scale is applied to each feature)
**X**	The coordinates of the individuals on the principal components.

**Step 5 Classification.** After Step 4, four classifiers, including naïve Bayes, maximum entropy, SVM, and random forest, are applied to train the processed data for classifying the text into classes. This study set all parameters at default values for the four classifiers and used 10 times random sampling and ten-fold cross validation to verify accuracy. The detailed description and settings are shown in Table 6.

**Table 6:** Classifier parameter settings.

**Naïve Bayes**	laplace	Positive double controlling Laplace smoothing. The default (0) disables Laplace smoothing.
	na.action	A function to specify the action to be taken if NAs are found. The default action is not to count them for the computation of the probability factors.
**ME**	use_sgd	A logical indicating that SGD parameter estimation should be used. Defaults to FALSE.
	verbose	Logical specifying whether to provide descriptive output about the training process. Defaults to FALSE, or no output.
**SVM**	scale	A logical vector indicating the variables to be scaled. Per default, data are scaled internally (both x and y variables) to zero mean and unit variance.
	type	C-classification
	kernel	Linear for two label. Radial basis for multiple label.
	gamma	Parameter needed for all kernels except linear (default: 1/(data dimension))
	cost	Cost of constraints violation (default: 1)
	tolerance	Tolerance of termination criterion (default: 0.001)
	epsilon	Epsilon in the insensitive-loss function (default: 0.1)
	shrinking	Option whether to use the shrinking-heuristics (default: TRUE)
**RF**	cutoff	A vector of length equal to number of classes. The ‘winning’ class for an observation is the one with the maximum ratio of proportion of votes to cutoff. Default is 1/k where k is the number of classes.

**Step 6 Evaluation.** This step utilizes accuracy to evaluate classification performance, the accuracy is calculated using a classified confusion matrix (as Table 7) for document-level sentiment classification with positive and negative labels [9]. The equations of accuracy is shown as Eq (1), because the experimental dataset has marked positive and negative sentiment reviews, this study based on confusion matrix to compute the accuracy by using Eq (1).

$$accuracy=\frac{True\;Positive+True\;negative}{All\;the\;document}$$ (1)

**Table 7:** Confusion matrix for sentiment classification.

	Predicted	
	Positive	Negative
Actual positive	True Positive	False Negative
Actual negative	False Positive	True Negative

## Results and discussion

Based on the proposed algorithm, this study collects two open datasets and utilizes different experimental modules to conduct the experiments and compare the results with the listing methods. The datasets are collected from websites which are widely used in text classification areas. The two datasets are a movie review dataset and a dataset of cardiovascular disease abstracts (OHSUMED). The detailed properties of the Movie and the OHSUMED datasets are shown in Table 8.

**Table 8:** Properties of the Movie and OHSUMED datasets.

	Movie dataset	Ohsumed dataset
**No. of review**	2000	6087
**Class**	Positive (1000): Negative(1000)	C02(391): C10(1562) C11(364): C14(2550) C20(1220)
**Feature**	Unigram	Unigram
**No. of attributes**	46467, 4 addition attributes	29385, 4 addition attributes
**Vector space**	2000*46467	6087*29385

### Movie review dataset

Based on different parameter settings of the TF-IDF, this study employs stemming to obtain different features, designs five module experiments to compare with the listing methods and discusses what factors will affect the classifier accuracy. The five module experiments have different settings and features, as shown in Table 9. After step 2 and step 3 of the proposed algorithm, the feature set has 46467 attributes. To test the effect of different settings, the “SVD then PCA” method is compared with the listing methods. The different settings are 10 times random sampling and ten-fold cross validation to test the performance.

**Table 9:** Experimental module (Movie dataset).

ID	Parameter	Number of features	
		NoStemming	Stemming
**Module 1**	All features	46467	30588
**Module 2**	MinDocfreq = 2	12346	9747
**Module 3**	MinDocfreq = 3	5966	5442
**Module 4**	RemoveSparseTerm = 0.99	4366	3667
**Module 5**	RemoveSparseTerm = 0.95	963	1048

**Fig 2:**
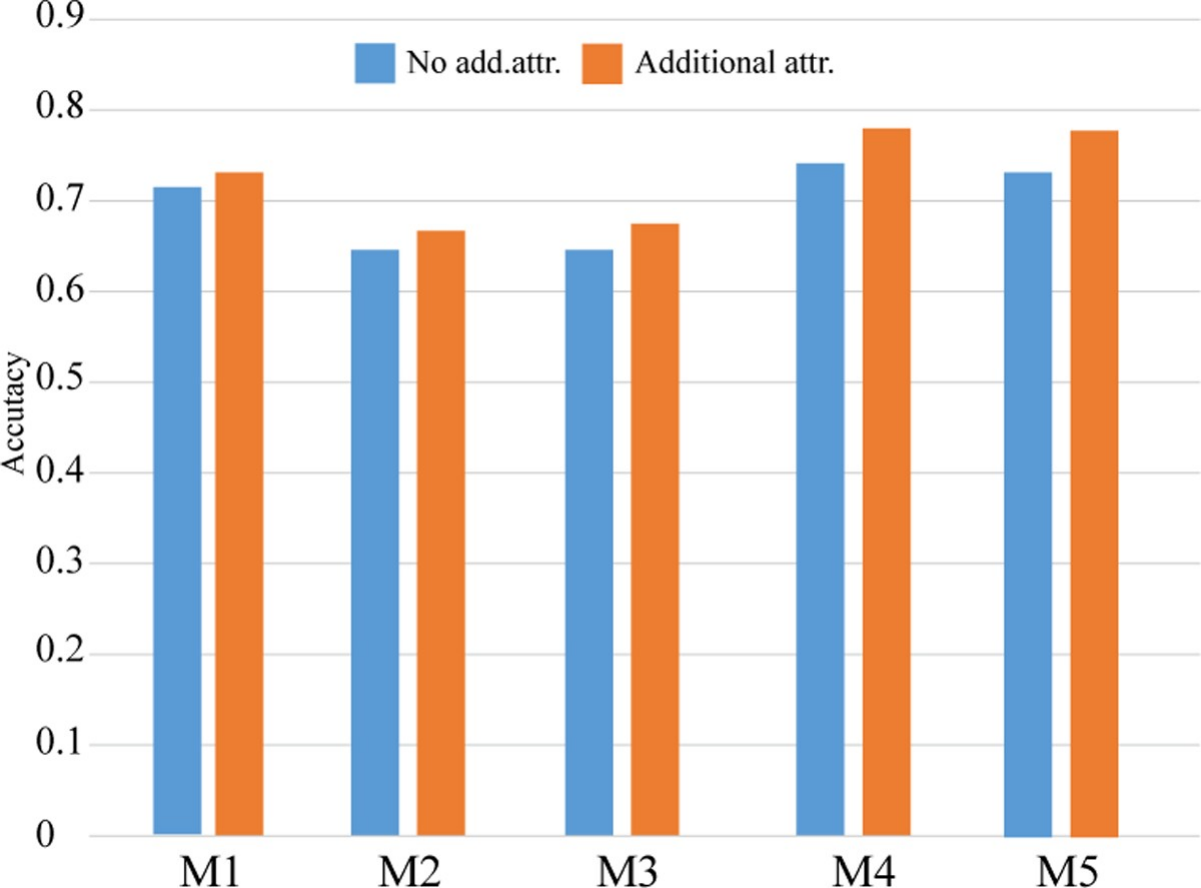
The effect of additional attributes for different modules (Movie dataset).

Table 10 shows that the proposed method with additional attributes is better than without additional attributes in terms of the average accuracy of the five classifiers; the SVM-linear and ME methods are better than the other classifiers in terms of accuracy. Table 11 (reduced dimension) shows the comparison results between with and without dimension reduction for Module 1 and Module 4 under no stemming. Overall, the proposed method with additional attributes is better than without additional attributes for both with and without dimension reduction. The SVM and ME classifiers are more accurate in most settings. From the experimental results, there are three findings as follows:

1. Feature extraction: The proposed method performs best on Module 1 and Module 4, as shown in Table 10. Module 4 obtains the highest accuracy in all of the experiments, and the number of features reduces to 9.4% (4366/46467 in no stemming of Table 9). The effect of stemming is not evident in this experiment, as shown in Table 10.
2. Additional attributes: To test the effect of additional attributes, we utilize the average of the five classifiers for Module 1 to Module 5, as shown in Table 10 and Fig 2. After the additional attributes are combined into the feature set, the results show that the additional attributes can obtain better results, especially with the SVM-RBF method.

**Table 10:** Comparison of results without dimension reduction (Movie dataset).

	Stem	Add Attr.	SVM-linear	SVM-RBF	NB	ME	RF	Ave.
**M1**	No	No	0.8437	0.4988	0.6151	0.8425	0.8133	0.7227
		Yes	0.8449	0.5434	0.6143	0.8440	0.8218	**0.7337**
	Yes	No	0.8379	0.5380	0.6226	0.8402	0.8139	0.7305
		Yes	0.8396	0.5396	0.6158	0.8412	0.8187	**0.7310**
**M2**	No	No	0.7401	0.5015	0.5018	0.7480	0.7361	0.6455
		Yes	0.7570	0.5514	0.4942	0.7601	0.7485	**0.6622**
	Yes	No	0.7366	0.5419	0.5007	0.7491	0.7352	0.6527
		Yes	0.7537	0.5912	0.5016	0.7719	0.7557	**0.6748**
**M3**	No	No	0.6736	0.6879	0.4981	0.6825	0.6646	0.6413
		Yes	0.7090	0.7361	0.4997	0.7166	0.7221	**0.6767**
	Yes	No	0.6648	0.6426	0.4983	0.6685	0.6921	0.6333
		Yes	0.7155	0.7346	0.4990	0.7161	0.7243	**0.6779**
**M4**	No	No	0.8401	0.5289	0.7242	0.8590	0.8211	0.7547
		Yes	0.8373	0.6627	0.7251	0.8513	0.8196	**0.7792**
	Yes	No	0.8178	0.5570	0.7259	0.8385	0.8190	0.7516
		Yes	0.8242	0.6703	0.7193	0.8417	0.8264	**0.7764**
**M5**	No	No	0.8194	0.5389	0.7440	0.7907	0.8142	0.7414
		Yes	0.8182	0.7070	0.7437	0.8147	0.8106	**0.7788**
	Yes	No	0.8032	0.5365	0.7479	0.8020	0.8115	0.7402
		Yes	0.8234	0.7064	0.7575	0.8216	0.8107	**0.7839**

**Table 11:** Dimension reduction results (Movie dataset).

	RD	Add Attr.	SVM-linear	SVM-RBF	NB	ME	RF	Ave.
**M1**	No	No	0.8437	0.4988	0.6151	0.8425	0.8133	0.7227
		Yes	0.8450	0.5434	0.6143	0.8440	0.8218	**0.7337**
	Yes	No	0.8452	0.5010	0.5308	0.8354	0.7190	0.6863
		Yes	0.8461	0.4890	0.5301	0.8397	0.7383	**0.6886**
**M4**	No	No	0.8401	0.5289	0.7242	0.8590	0.8211	0.7547
		Yes	0.8373	0.6627	0.7251	0.8513	0.8196	**0.7792**
	Yes	No	0.8279	0.8273	0.5348	0.8274	0.7077	0.7450
		Yes	0.8383	0.8320	0.5234	0.8389	0.7124	**0.7490**
3. Dimension reduction: The results show that the accuracy with dimension reduction approaches the accuracy without dimension reduction, as shown in Table 11. The proposed additional attributes obtain a better accuracy in the Movie dataset. Therefore, the additional attributes and the “SVD then PCA” methods can enhance the performance in sentimental classification.

### OHSUMED dataset

The second dataset is collected from the OHSUMED corpus, which was created by Hersh et al. [35–36]. The dataset has 50216 documents in 23 categories as Table 2. The classification considered here is a multiple class classification that classifies the documents as class C02, 10, 11, 14, and 20. The full feature set contains 29385 attributes. To test the effect of different feature extraction settings, Table 8 shows the properties of the OHSUMED datasets, and Table 12 shows that the five module experiments have different settings and features for ohsumed dataset. Next, the SVD and PCA are employed with each configuration of settings, so that the effect of dimension reduction can be measured. Finally, the different settings are randomly divided into 10 groups for cross validation.

**Table 12:** Experimental module (Ohsumed dataset).

ID	Parameter	Number of features	
		No Stemming	Stemming
Module 1	Full features	29385	21911
Module 2	MinDocfreq = 2	13694	11131
Module 3	MinDocfreq = 3	8371	7207
Module 4	RemoveSparseTerm = 0.99	1394	1226
Module 5	RemoveSparseTerm = 0.95	226	258

**Fig 3:**
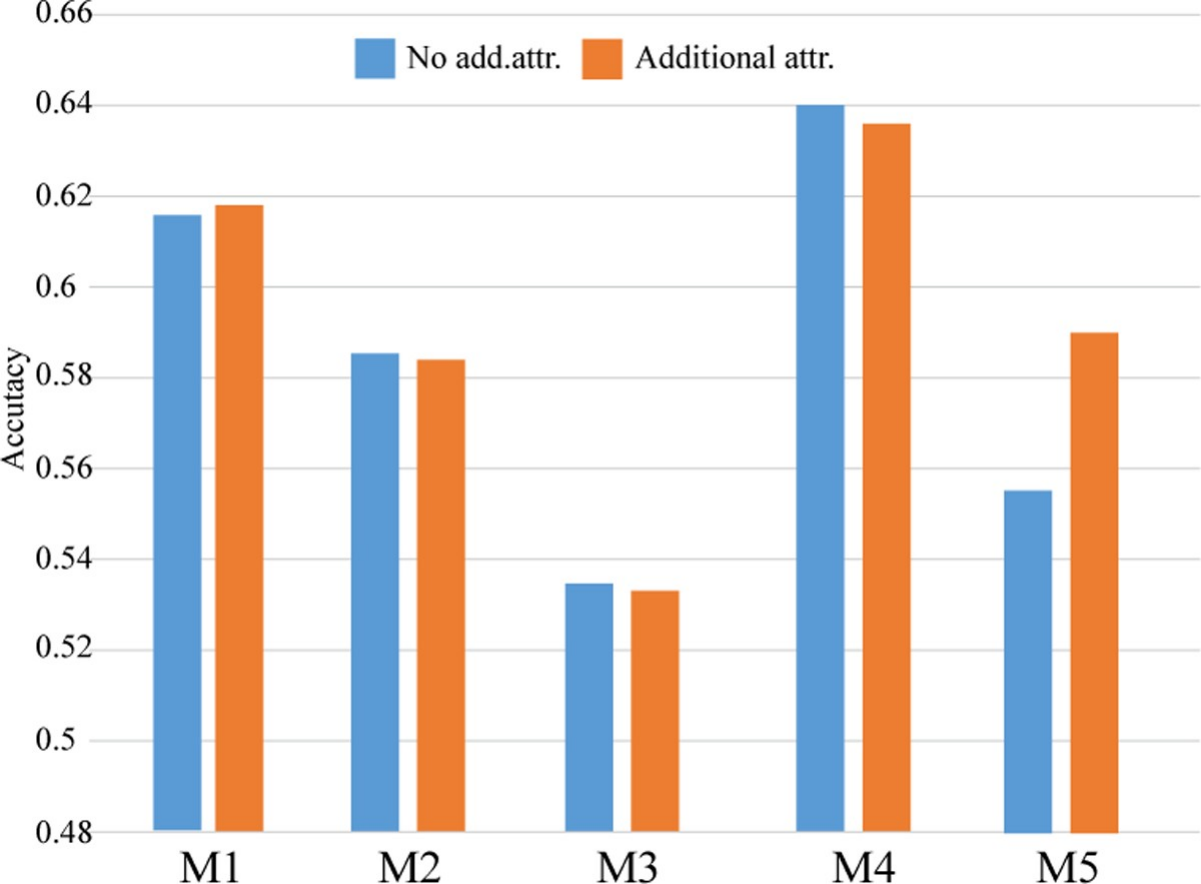
The effect of additional attributes on the different modules (OHSUMED dataset).

Table 13 (without dimension reduction), Table 14 (with dimension reduction), and Fig 3 show the results of the OHSUMED dataset. From Table 13, the best experimental models are Module 1 and Module 4. Overall, the effect of stemming is still not obvious. For the same reason described for the Movie dataset, Table 14 shows the results of Module 1 and Module 4 for dimension reduction for comparison. Overall, Module 1 achieved the highest accuracy of 0.7879 without dimension reduction and achieved an accuracy of 0.7126 with dimension reduction. The SVM with RBF kernel method has the best accuracy when the features are large.

**Table 13:** Results of the OHSUMED dataset without dimension reduction.

			SVM-linear	SVM-RBF	NB	ME	Ave
**M1**	**NoStem**	**NoAtt**	0.7408	0.7874	0.0791	0.7440	0.6133
		**YesAtt**	0.7493	0.7937	0.0770	0.7372	**0.6152**
	**Stem**	**NoAtt**	0.7401	0.7879	0.0787	0.7412	0.6123
		**YesAtt**	0.7381	0.7948	0.0803	0.7483	**0.6140**
**M2**	**NoStem**	**NoAtt**	0.7116	0.7625	0.0606	0.6996	**0.5884**
		**YesAtt**	0.7217	0.7596	0.0606	0.6974	0.5872
	**Stem**	**NoAtt**	0.7128	0.7692	0.0661	0.6954	**0.5889**
		**YesAtt**	0.7139	0.7746	0.0656	0.6950	0.5903
**M3**	**NoStem**	**NoAtt**	0.6273	0.7085	0.0569	0.6193	**0.5342**
		**YesAtt**	0.6286	0.7158	0.0569	0.5980	0.5334
	**Stem**	**NoAtt**	0.6369	0.7212	0.0589	0.6249	**0.5423**
		**YesAtt**	0.6185	0.7252	0.0589	0.6111	0.5335
**M4**	**NoStem**	**NoAtt**	0.6726	0.7661	0.4340	0.6168	**0.6396**
		**YesAtt**	0.6736	0.7532	0.4339	0.6114	0.6359
	**Stem**	**NoAtt**	0.6757	0.7702	0.4460	0.6173	0.6445
		**YesAtt**	0.6772	0.7788	0.4457	0.6107	**0.6448**
**M5**	**NoStem**	**NoAtt**	0.4331	0.6096	0.4710	0.6299	0.5524
		**YesAtt**	0.5962	0.6408	0.4732	0.6301	**0.5910**
	**Stem**	**NoAtt**	0.6132	0.6619	0.4821	0.6417	0.6033
		**YesAtt**	0.6153	0.6643	0.4837	0.6424	**0.6047**

**Table 14:** Results of the OHSUMED dataset with dimension reduction.

			SVM-linear	SVM-RBF	NB	ME	RF	Ave
**M1**	**NoRD**	**NoAtt**	0.7408	0.7874	0.0791	0.7440	0.7150	0.6133
		**YesAtt**	0.7493	0.7877	0.0770	0.7372	0.7186	**0.6140**
	**YesRD**	**NoAtt**	0.7082	0.2629	0.3155	0.5265	0.6063	**0.4839**
		**YesAtt**	0.7127	0.2816	0.3128	0.5378	0.6204	0.4931
**M4**	**NoRD**	**NoAtt**	0.6726	0.7621	0.4340	0.6168	0.7086	**0.6388**
		**YesAtt**	0.6736	0.7632	0.4339	0.6114	0.7073	0.6378
	**YesRD**	**NoAtt**	0.6489	0.6835	0.4141	0.5715	0.4847	0.5605
		**YesAtt**	0.6502	0.6883	0.4103	0.5852	0.4923	**0.5653**

RD = Reducing dimension

### Findings

From the experimental results, some findings are summarized as follows:

1. **Attribute extraction:** From Table 10 and Fig 2, the results of the Movie dataset show that Module 4 and Module 5 are better than the other modules. Module 4 achieves a higher accuracy in the overall experiments, and the number of attributes is decreased to 9.4% (4366/46467 in no stemming of Table 9). Furthermore, Table 10 shows that the effect of stemming is not obvious in the experiments. In the OHSUMED dataset, as shown in Table 13 and Fig 3, Module 1 and Module 4 are better than the other modules. Module 4 obtains a higher accuracy in the overall experiments, and the number of attributes is reduced to 5.6% (1226/21911 in stemming of Table 12). Therefore, we find that Module 4 shows effects from stemming, which means that stemming can reduce the number of attributes and increase the computational speed.
2. **Adding additional attributes:** This study proposes adding additional features to improve the accuracy of text classification. *i*.*e*., increasing the frequency of positive and negative adjectives, and adverbs. In the Movie dataset, to test the impact of adding additional attributes, this study calculates the average accuracy of five classifiers from Module 1 to Module 5, and the results are shown in Table 10. Table 10 and Fig 3 show that adding additional attributes can increase the accuracy, especially for the SVM_RBF classifier. In the OHSUMED dataset, see Table 13 and Fig 3, the best experimental models are obtained with Module 1 and Module 4, and the effect of stemming is slightly improved in terms of average accuracy. In addition, Fig 3 shows that without stemming, Module 1 to Module 5 have better performance with additional attributes than without additional attributes.
3. **Dimension reduction:** From the Movie and OHSUMED dataset experiments, Tables 11 and 14 show the results with and without dimension reduction, and the accuracy with dimension reduction is close to the accuracy without dimension reduction. After dimension reduction, the proposed method with additional attributes can obtain better results in the Movie dataset. To test the “SVD then PCA” method could shorten the implementation time in sentimental text mining, the two experimental datasets were implemented in R (R-3.2.1 version) on an Intel i7-3770k, 3.5 GHz CPU, Microsoft Windows 10 system. The total implementation time of five classifier is listed in Table 15, among five modules, four modules can reduce the total implementation time except Module 5. Therefore, adding additional attributes and dimension reduction are feasible for the proposed method.

**Table 15:** The total implementation time of five classifiers (time unit: Second).

			Movie dataset		Ohsumed dataset	
			No SVD then PCA	SVD then PCA	No SVD then PCA	SVD then PCA
M1	NoStem	NoAtt	69470.01	**4548.81**	255703.80	**21389.70**
		YesAtt	69994.24	**4392.11**	255991.03	**20485.60**
	Stem	NoAtt	46015.38	**4701.47**	218711.62	**22891.20**
		YesAtt	37909.68	**4854.29**	210774.51	**21857.15**
M2	NoStem	NoAtt	25483.73	**4688.52**	290414.11	**20186.34**
		YesAtt	22561.66	**4736.13**	271452.64	**18944.52**
	Stem	NoAtt	17607.3	**4652.27**	212699.18	**18609.08**
		YesAtt	15987.36	**4750.66**	203012.15	**19002.64**
M3	NoStem	NoAtt	22372.52	**4570.21**	191078.71	**13710.63**
		YesAtt	12381.92	**4429.86**	182596.45	**13289.58**
	Stem	NoAtt	12727.8	**4845.42**	206772.09	**14536.26**
		YesAtt	10859.73	**4816.42**	193875.12	**14449.26**
M4	NoStem	NoAtt	7215.96	**4416.17**	57854.56	**13248.51**
		YesAtt	7223.3	**4521.94**	60521.87	**13565.82**
	Stem	NoAtt	4603.17	**4595.66**	14235.25	**13786.98**
		YesAtt	4568.9	4726.66	13458.21	14179.98
M5	NoStem	NoAtt	1412.23	1813.43	3985.27	7253.72
		YesAtt	1385.44	1905.84	4125.84	7623.36
	Stem	NoAtt	1564.86	2177.75	1356.85	8711.00
		YesAtt	1513.7	2340.69	1421.54	9362.76

(1) The bold denotes the “SVD then PCA” method can reduce implementing time; (2) The experiment was implemented in R (R-3.2.1 version) on an Intel i7-3770k, 3.5 GHz CPU, Microsoft Windows 10 system.

## Conclusions

This study proposed an additional feature method to enhance accuracy and the “SVD then PCA” method to shorten the implementation time in sentimental text mining. The additional features are frequencies of positive and negative adjectives and adverbs. The results of two experiments show that the proposed method can obtain better accuracy than other methods, and adding additional attributes can increase the accuracy, especially for the SVM_RBF classifier. In terms of the classifier, the SVM and ME are shown to be the best choice for sentiment classification. In the future, there are still several issues that can be examined as an extension of this study as follows:

1. In the feature selection method, the following issues can be discussed: (i) use a domain specific lexicon to find or filter features, (ii) assign different weights to features to improve accuracy, and (iii) consider the relationships between words and documents.
2. Apply the proposed method to different application fields, such as reputation monitoring and social emotion detection.

## Supporting information

S1 Dataset Text mining datasets. (ZIP)
